# Development of a Machine Learning Model for Optimal Applicator Selection in High-Dose-Rate Cervical Brachytherapy

**DOI:** 10.3389/fonc.2021.611437

**Published:** 2021-03-05

**Authors:** Kailyn Stenhouse, Michael Roumeliotis, Philip Ciunkiewicz, Robyn Banerjee, Svetlana Yanushkevich, Philip McGeachy

**Affiliations:** ^1^ Department of Physics and Astronomy, University of Calgary, Calgary, AB, Canada; ^2^ Department of Medical Physics, Tom Baker Cancer Centre, Calgary, AB, Canada; ^3^ Department of Oncology, University of Calgary, Calgary, AB, Canada; ^4^ Department of Biomedical Engineering, University of Calgary, Calgary, AB, Canada; ^5^ Department of Radiation Oncology, Tom Baker Cancer Centre, Calgary, AB, Canada; ^6^ Department of Electrical and Computer Engineering, University of Calgary, Calgary, AB, Canada

**Keywords:** gynecologic brachytherapy, intracavitary brachytherapy (ICBT), high-dose-rate brachytherapy, radiation oncology, machine learning, decision-support tools

## Abstract

**Purpose:**

To develop and validate a preliminary machine learning (ML) model aiding in the selection of intracavitary (IC) versus hybrid interstitial (IS) applicators for high-dose-rate (HDR) cervical brachytherapy.

**Methods:**

From a dataset of 233 treatments using IC or IS applicators, a set of geometric features of the structure set were extracted, including the volumes of OARs (bladder, rectum, sigmoid colon) and HR-CTV, proximity of OARs to the HR-CTV, mean and maximum lateral and vertical HR-CTV extent, and offset of the HR-CTV centre-of-mass from the applicator tandem axis. Feature selection using an ANOVA F-test and mutual information removed uninformative features from this set. Twelve classification algorithms were trained and tested over 100 iterations to determine the highest performing individual models through nested 5-fold cross-validation. Three models with the highest accuracy were combined using soft voting to form the final model. This model was trained and tested over 1,000 iterations, during which the relative importance of each feature in the applicator selection process was determined.

**Results:**

Feature selection indicated that the mean and maximum lateral and vertical extent, volume, and axis offset of the HR-CTV were the most informative features and were thus provided to the ML models. Relative feature importances indicated that the HR-CTV volume and mean lateral extent were most important for applicator selection. From the comparison of the individual classification algorithms, it was found that the highest performing algorithms were tree-based ensemble methods – AdaBoost Classifier (ABC), Gradient Boosting Classifier (GBC), and Random Forest Classifier (RFC). The accuracy of the individual models was compared to the voting model for 100 iterations (ABC = 91.6 ± 3.1%, GBC = 90.4 ± 4.1%, RFC = 89.5 ± 4.0%, Voting Model = 92.2 ± 1.8%) and the voting model was found to have superior accuracy. Over the final 1,000 evaluation iterations, the final voting model demonstrated a high predictive accuracy (91.5 ± 0.9%) and F1 Score (90.6 ± 1.1%).

**Conclusion:**

The presented model demonstrates high discriminative performance, highlighting the potential for utilization in informing applicator selection prospectively following further clinical validation.

## Introduction

Globally, cervical cancer is the fourth most commonly diagnosed cancer and the fourth leading cause of cancer death in women (1, 2). For patients with locally advanced disease - International Federation of Gynecology and Obstetrics (FIGO) stage IB2-IVA - the current standard of care includes pelvic external beam radiotherapy (EBRT) with concurrent chemotherapy plus a brachytherapy boost (2–6). High-dose-rate (HDR) brachytherapy is crucial in the treatment of locally advanced cervical cancer (LACC). Due to its high dose conformity and rapid dose fall-off outside of the target region, brachytherapy is able to deliver a higher dose of radiation to the tumour volume while providing superior normal tissue sparing when compared to external beam techniques (7–9). Several studies have shown that utilizing brachytherapy in addition to EBRT improves survival rates and local control in patients with LACC (4, 8, 10).

HDR brachytherapy treatments use an applicator, placed through the vaginal cavity that treats the upper vagina, cervix, and uterus (3). The most commonly used intracavitary (IC) applicators are the tandem and ovoid and ring and tandem (3). Additionally, hybrid intracavitary-interstitial (IS) applicators have been developed that allow for the use of interstitial catheters that are guided by the ring or ovoid, once positioned in the patient (6, 11, 12).

One challenge inherent to this procedure is the selection of the applicator, as different applicator geometries result in a different dose distribution (11). Some recommendations exist to guide applicator selection – current literature identifies a lateral extent of the high-risk clinical target volume (HR-CTV) > 25 mm and an HR-CTV volume > 30 cm^3^ as indicators for the use of interstitial needles (13, 14). However, the decision is still largely dependent on the physician’s judgement and experience (3). Sub-optimal applicator selection may arise, resulting in sub-par dose distributions. This can decrease the probability of providing local control to the tumour as well as delivering unwanted dose to surrounding healthy organs, leading to less-desirable patient outcomes (15–17).

Machine learning (ML) has been applied in radiotherapy to act as an expert decision-support tool which has seen increased attention in medical physics in recent years (18). ML techniques have seen considerable interest in radiation oncology, with applications in brachytherapy ranging from treatment planning to automatic low-dose-rate (LDR) seed detection (19–22). In recent years, there has been an increased focus on utilizing ML to improve HDR cervical brachytherapy. Recent work has included predicting fistula formation for patients receiving interstitial gynecologic brachytherapy (23), ML-aided automatic digitization of interstitial needles and applicators (24, 25), and reinforcement-learning based inverse treatment planning for tandem-and-ovoid brachytherapy (26). Despite this research, the extent to which ML can be used to support the applicator selection process for cervical HDR brachytherapy has not been explored.

The ability of ML models to mimic human modes of thinking in highly complex reasoning tasks makes them well suited to provide decision-support for the applicator selection process (27). Additionally, ML models are able to identify features important for applicator selection and weigh their contributions to make an informed applicator selection, while accounting for historical physician experience in the form of training data. This could provide planning assistance to brachytherapy departments by increasing the uniformity in applicator selection and providing support to less experienced clinicians. This work describes a rigorous methodology used in the development of an ML model, including algorithm selection, model optimization, feature selection, and model performance evaluation using clinical patient data. The work presented is a necessary validation step demonstrating the ability for ML to be used as a decision-support tool in the applicator selection process. In its current state, the model can provide external validation for a physician’s selection of applicator post-insertion. This preliminary validation will allow for future expansion of the model to include predictions for optimal needle arrangements for treatments requiring interstitial needles and for future use in prospective applicator selection for HDR cervical brachytherapy following clinical validation. A prospective study utilizing the presented ML model on pre-insertion geometry metrics has been approved to evaluate true prospective applicator selection.

## Materials and Methods

### Patient Cohort

Data for the ML model was extracted from our institutional cervical HDR brachytherapy patient database. Treatment data for patients treated between 2015 and 2020 was compiled, which included 233 treatment fractions (147 IC, 86 IS) for 83 patients. The patients in this cohort were treated with one of three applicator types: (i) tandem and ovoid (IC), (ii) ring and tandem (IC), or (iii) ring and tandem with the additional of interstitial needles (IS). In this work, the two intracavitary applicator types were grouped to form one class (IC), with the hybrid interstitial applicator forming the other class (IS). Oncentra^®^ Brachy TPS version 3.3 (Nucletron, Elekta AB, Stockholm, Sweden) was used for treatment planning for all cases to deliver a prescription dose of 8 Gy x 3 fractions to the HR-CTV with each fraction separated by a week. The separation of treatments allows for the independent selection of applicator for each fraction. For patients prescribed 8 Gy x 3 fractions who were unable to complete the treatment course, any completed fractions were included in the data set.

### Model Framework

**Figure 1:**
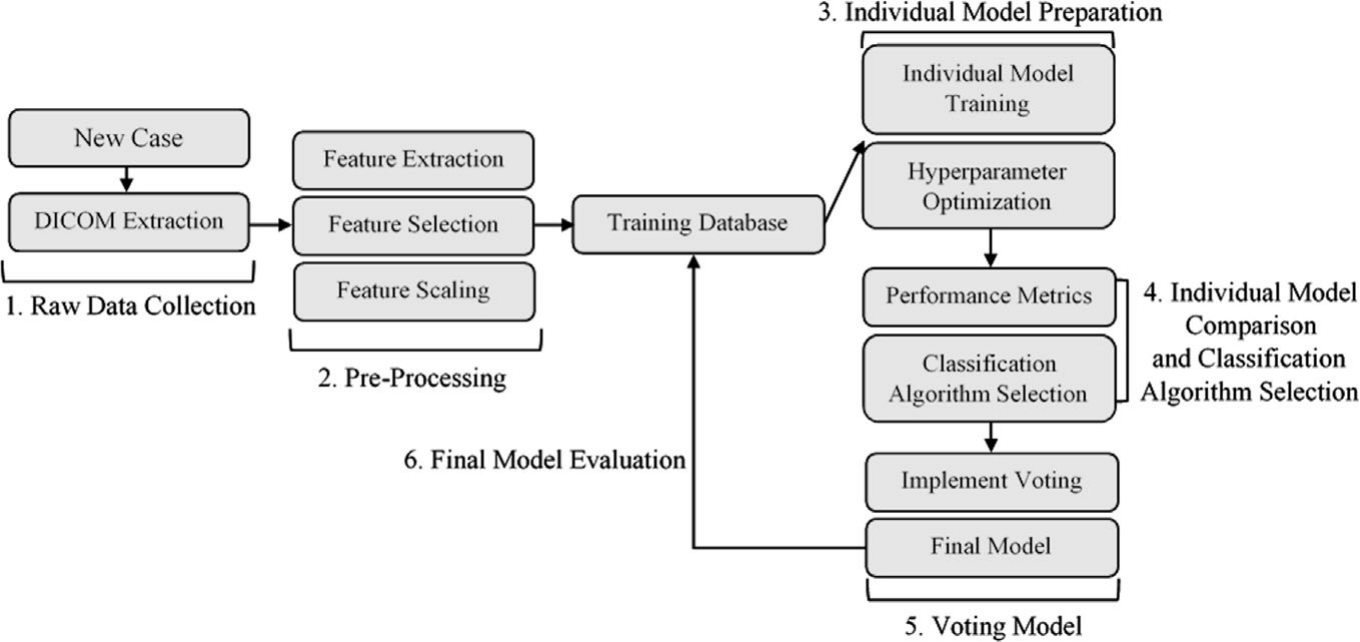
Model development workflow. (1) Data collection and DICOM extraction. (2) Data pre-processing and feature engineering. (3) Training and optimization of a set of classification algorithms for comparison. (4) Selection of subset of trained and optimized individual models. (5) Individual model combination through voting. (6) Evaluation of final ML voting model. DICOM, Digital Imaging and Communications in Medicine.

**Figure 1** illustrates the workflow used to develop the applicator prediction ML model, broken down into six major development steps.

### Data Preprocessing and Training Database

For each fraction, structure, plan, and dose data files were extracted from the Oncentra^®^ Brachy TPS in Digital Imaging and Communications in Medicine (DICOM) format. Applicator type, interstitial needle patterns, and relevant structure contours (HR-CTV, intermediate-risk clinical target volume (IR-CTV), gross tumour volume (GTV), bladder, rectum, sigmoid) were extracted and stored within the training database. For the first treatment fraction, contours were derived from post-insertion MR images. Subsequent treatment fractions derived contours from post-insertion CT images, using previous MR images to aid in the delineation of the HR-CTV. To account for class imbalance in the dataset (147 IC fractions, 86 IS fractions), random oversampling from the minority applicator class was employed to balance the classes. In consultation with a clinical brachytherapy team, the features extracted for this investigation were those expressing the geometric characteristics and relationships between the target volume, organs-at-risk (OARs), and the applicator.

**Figure 2:**
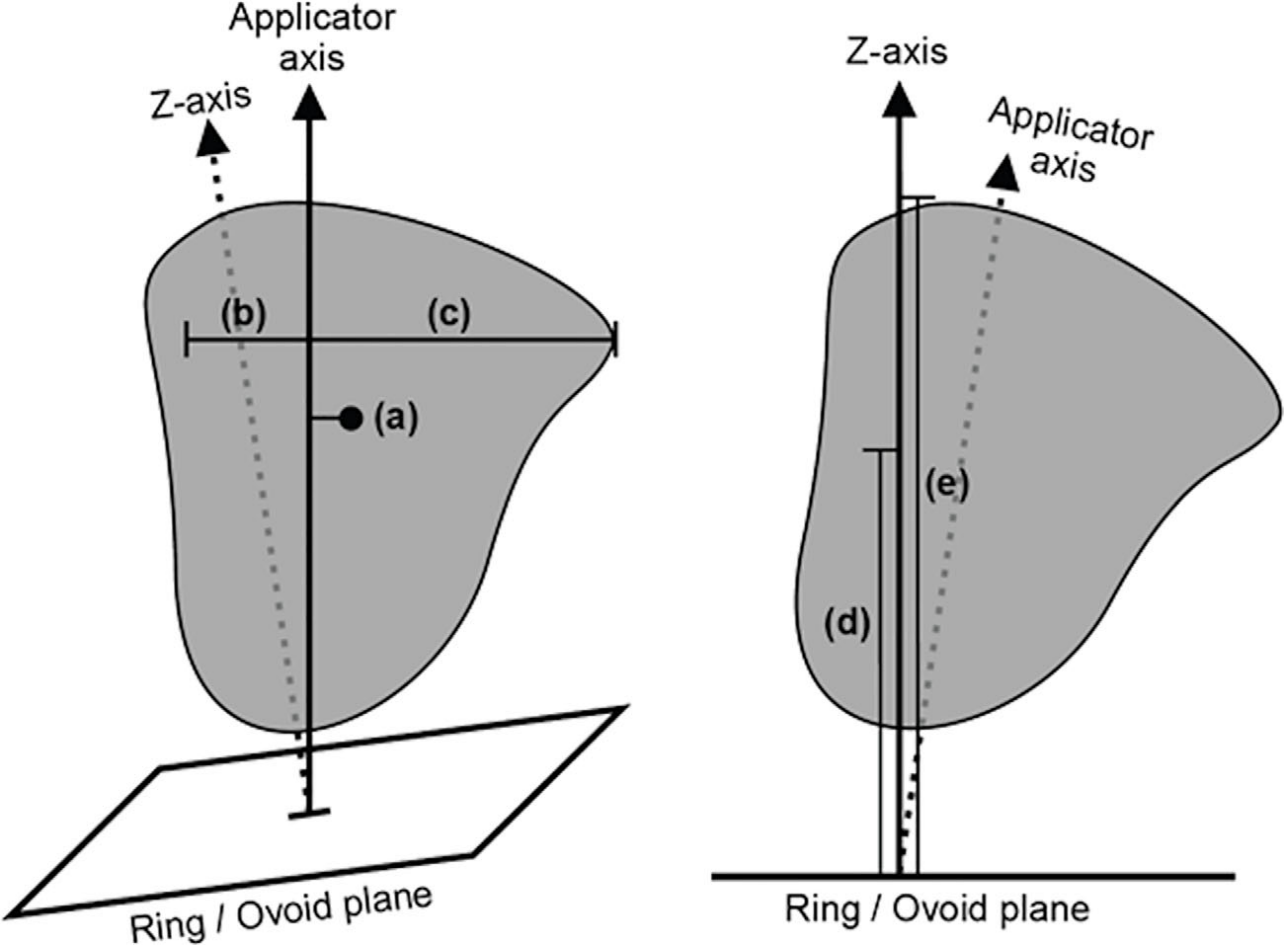
A diagram illustrating the selected high-risk clinical target volume (HR-CTV) spread metrics, as defined relative to the applicator geometry. The illustrated metrics are (a) axis offset between the centre-of-mass of the HR-CTV and the applicator tandem axis, (b) mean and (c) maximum lateral extent of the HR-CTV orthogonal to the applicator tandem axis, and (d) mean and (e) maximum vertical extent of the HR-CTV orthogonal to the ring/ovoid plane.

From the structure contours, a series of lower-dimensional geometry metrics were calculated, which were expected to indicate how well an applicator type would be able to achieve optimal dosimetry. The geometry metrics were structure volumes, proximity of the OARs to the HR-CTV, and geometric characteristics of the HR-CTV. Structure volumes were extracted for the bladder, rectum, sigmoid colon, and HR-CTV. The proximity of the OARs to the HR-CTV was defined as the mean distance between the voxels of the HR-CTV and the nearest 1.5 cc of the respective OARs (bladder, rectum, and sigmoid colon). The geometric characteristics of the HR-CTV were the mean and maximum lateral extent of the HR-CTV (orthogonal to the tandem axis), the mean and maximum vertical extent of the HR-CTV (orthogonal to the ring or ovoid plane), and the axis offset between the centre-of-mass of the HR-CTV and the tandem axis of the applicator, which is an indicator of asymmetry in the HR-CTV geometry. These HR-CTV geometry metrics are illustrated in **Figure 2**.

Each treatment fraction was assigned a weighting – to be utilized during the training of the ML model – according to the fraction’s adherence to EMBRACE dosimetric quality metrics (D90 HR-CTV, D98 HR-CTV, D98 GTV, D98 IR-CTV, Point A EQD2, Bladder D2cc, Rectum D2cc, Sigmoid D2cc) (28). This was implemented to account for possible sub-optimal applicator selection in the training data. A fraction with poor dosimetric outcomes would thus be used less strongly in the training of the ML model. The decimal weighting assigned to each treatment fraction based on its dosimetric quality is outlined in **Table 1**.

**Table 1:** Treatment fraction data weighting regime utilized in the model training process and the number of data points belonging to each category.

Weighting Assigned	# Planning Aim Violations	# Dose Constraint Violations	# Fractions
1.0	≤2	None	134
0.5	>2	None	32
0.25	Any Number	≥1	67

Weights were assigned on a per-fraction basis based on adherence to EMBRACE dosimetric quality metrics (D90 HR-CTV, D98 HR-CTV, D98 GTV, D98 IR-CTV, Point A EQD2, Bladder D2cc, Rectum D2cc, Sigmoid D2cc) (28). The highest quality plans are assigned full weight, moderate quality plans are assigned half weight, and the lowest quality plans are assigned quarter weight.

### Feature Selection

To select important features for use in model training and evaluation, a series of univariate feature selection tests were performed. This was employed to prevent overwhelming the model with redundant or unimportant information that would reduce predictive capabilities First, the mutual information between the features and the target variable (i.e., the applicator class, IC or IS) was calculated. A higher mutual information score indicates that there is information about the target variable that can be obtained by knowing the feature. Second, an ANOVA F-test was performed. The F-test was used to determine the correlation between the input features and the output classification. A higher F-score indicates that there is a relationship between the value of a given feature and applicator choice. In both cases, features that will have the largest contribution to model performance can be selected by ranking mutual information and F-scores.

### Classification Algorithm Selection and Individual Model Optimization

Classification algorithm selection and model training, optimization and testing utilized the Scikit-Learn package for Python (29). Each of the 233 fractions was labelled with the use of either IC or IS applicators. Since the desired output of the model is a prediction of class (i.e., IS or IC applicator type), a series of supervised classification ML algorithms were investigated. In this work, the classification algorithms investigated were intended to be interpretable compared to more complex ML algorithms that act as a “black box”, which is important when dealing with limited data and models that can have potentially high variance (18, 30).

The initial subset of classification algorithms selected for training individual models are listed in **Table 2** with their utilized abbreviations and compatibility with using weighted samples during the training process. For classification algorithms that supported training with sample weights (see **Table 2**), the trained individual model performance was evaluated using both weighted and unweighted samples. To tune the individual models and compare performance, a process of nested 5-fold cross-validation and hyperparameter optimization was performed. This methodology is similar to that performed by Deist et al. (31).

**Table 2:** List of compared classification algorithms, their utilized abbreviation, and compatability with using weighted samples when training the individual model.

Classification Algorithm	Abbreviation	Weighted Samples?
AdaBoost Classifier	ABC	Yes
Gaussian Naïve Bayes Classifier	GNB	Yes
Gaussian Process Classifier	GPC	No
Gradient Boosting Classifier	GBC	Yes
K-Nearest Neighbours Classifier	KNN	No
Linear Discriminant Analysis	LDA	No
Logistic Regression Classifier	LRC	Yes
Multi-layer Perceptron Classifier	MLPC	No
Nearest Centroid Classifier	NCC	No
Nu-Support Vector Classifier	NuSVC	Yes
Quadratic Discriminant Analysis	QDA	No
Random Forest Classifier	RFC	Yes

**Figure 3:**
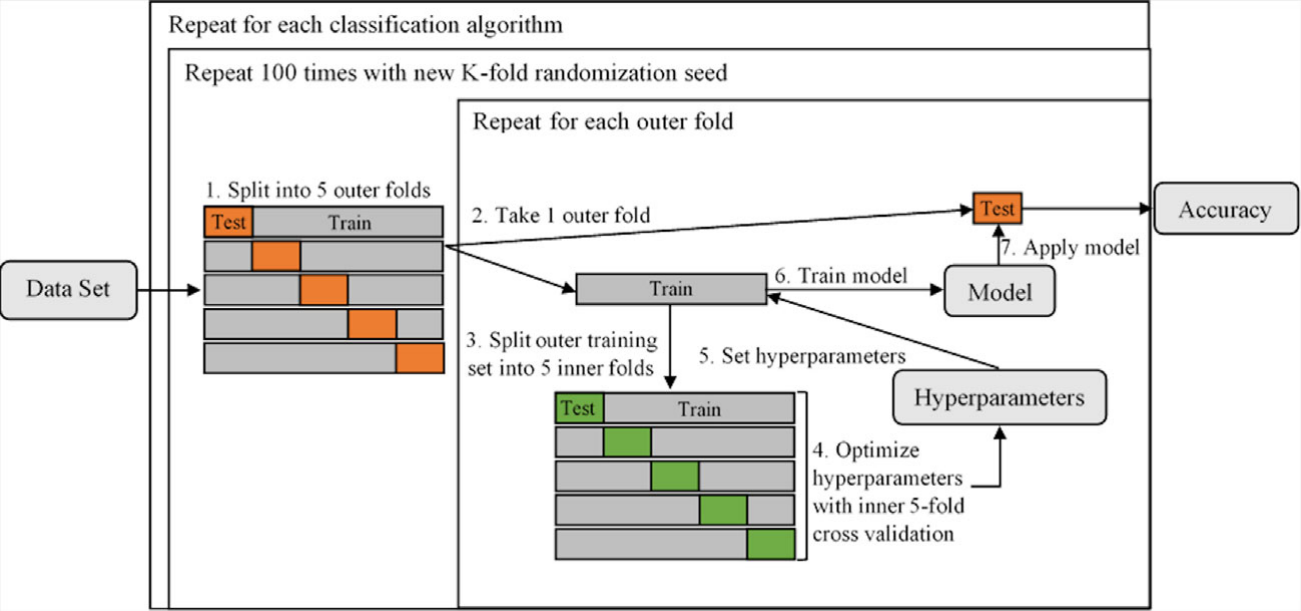
A diagram illustrating the process of tuning hyperparameters and determining individual model performance using nested 5-fold cross-validation. The dataset is first split into five outer folds (Step 1), each acting once as the test set and the remaining four times as a component of the training set. The training set is then split into five inner folds (Steps 2 and 3, repeated for each of the outer folds), on which the hyperparameters are tuned (Step 4). Both inner and outer 5-fold cross-validation utilized the KFold function in Scikit-Learn (parameters were set to n_splits = 5, shuffle = True, random_state = iteration number). The optimal hyperparameteres are then set (Step 5) and the individual model is trained on the outer training set (Step 6) and performance is evaluated on the outer test set (Step 7).

The process of hyperparameter tuning and individual model optimization is depicted in **Figure 3**. For 100 evaluation iterations of each individual model (**Table 2**), the full dataset was randomly split into five subsamples using the KFold function in Scikit-Learn (Step 1). Each subsample acted once as the test set and the remaining four times as a component of the training set. Following common practice, five folds were used for the inner and outer cross-validation (32). The individual models were trained on the training set and out-of-sample performance metrics were calculated on the test set, including accuracy, precision, recall, F1 score, and ROC – AUC (Receiver Operating Characteristics – Area Under the Curve). The use of five folds resulted in five estimates per performance metric, which were then averaged to assess the performance for each of the 100 iterations. While training using each outer fold, optimal hyperparameters were selected using a rigorous grid search using the GridSearchCV function in Scikit-Learn to maximize the accuracy of an inner five-fold cross-validation (Steps 3-6). For this inner cross-validation, the outer training set was again split into five random subsamples used to compare models with different hyperparameters using the KFold function. Classification algorithm selection and individual model optimization without this nested cross-validation uses the same data to tune the model parameters and evaluate model performance, causing information to leak into the model and cause overfitting of the data (33). A list of the hyperparameters that were optimized and their final optimized values for each classification algorithm is provided in Supporting Information **Table S1**.

Once the subset of individual models was trained, the top three highest performing were used to form the voting model. Accuracy - the ratio of correct classifications - was the metric selected for model comparison due to its interpretability and widespread use in performance evaluation. Three individual models were chosen to ensure that if volatility was present in one, two well-calibrated models would be in place to counteract it.

### Voting Model

The top three individual models selected during the model optimization process were then compiled into a voting model. The voting model works by placing equal weight on each of the three chosen individual models. Each model individually makes a class prediction of IC or IS and the final voted classification result is determined through a soft voting process. Soft voting predicts the class label based on the weighted average of the predicted probabilities, which is recommended for an ensemble of well-calibrated classifiers (34).

### Feature Importance for Individual Models in Voting Model

Feature importance was investigated for the individual models used in the final voting model. The relative importance of a feature is indicative of how often the model uses this feature to make key decisions. For the selected tree-based classification algorithms, the relative importance of a feature is a measure of how a given feature-based split point improves the classification accuracy. The importance of a feature is determined by the model during the training process.

### Voting Model Evaluation

The final voting model was assessed over 1,000 iterations of different stratified train (85%) and test (15%) data subsets. For each iteration, the random state was set to the iteration number. Additionally, a Leave-One-Out Cross-Validation (LOOCV) was performed wherein the test dataset contains only one data point, with the remaining data being used to train the model. LOOCV is a more practical estimate of model performance as it mimics how the model will perform when applied in a practical setting, as it will be provided with only one sample in the test set after training on the full training dataset. Additionally, in situations where model performance is limited by a small dataset, LOOCV can provide a better estimation of model performance as K-fold cross-validation may provide an overly pessimistic estimate (32).

## Results

### Feature Selection

The ANOVA F-score and mutual information were calculated for the full set of features, which are shown in **Table 3**. Both tests indicated that the three most informative features were the HR-CTV volume, and the mean and maximum lateral extent of the HR-CTV. Both tests also identified the proximity of the HR-CTV to the OARs were noisy, uninformative features. Based on these results, the set of features provided to the classification algorithms was halved to include only the geometric characteristics of the HR-CTV to reduce model complexity and remove features that did not demonstrate a strong correlation with applicator selection.

**Table 3:** ANOVA F-Score and mutual information metrics calculated for each of the extracted geometric features.

Feature Type	Feature	F-Score	Mutual Information
HR-CTV Geometry Metric	Axis Offset	49.47	0.19
	**Lateral Mean Extent**	**161.01**	**0.35**
	**Lateral Maximum Extent**	**219.92**	**0.32**
	Vertical Mean Extent	27.73	0.26
	Vertical Maximum Extent	61.44	0.24
	**Volume**	**120.46**	**0.35**
OAR Volume	Rectum Volume	3.60	0.05
	Bladder Volume	0.44	0.13
	Sigmoid Volume	1.04	0.07
OAR Proximity to HR-CTV	Rectum Proximity	12.67	0.10
	Bladder Proximity	16.48	0.19
	Sigmoid Proximity	0.08	0.08

These values were used to reduce the number of features used in the model by selecting only those thank ranked the highest under both metrics. The top three features for each test have been bolded for reference.

### Classification Algorithm Selection and Individual Model Optimization

**Figure 4:**
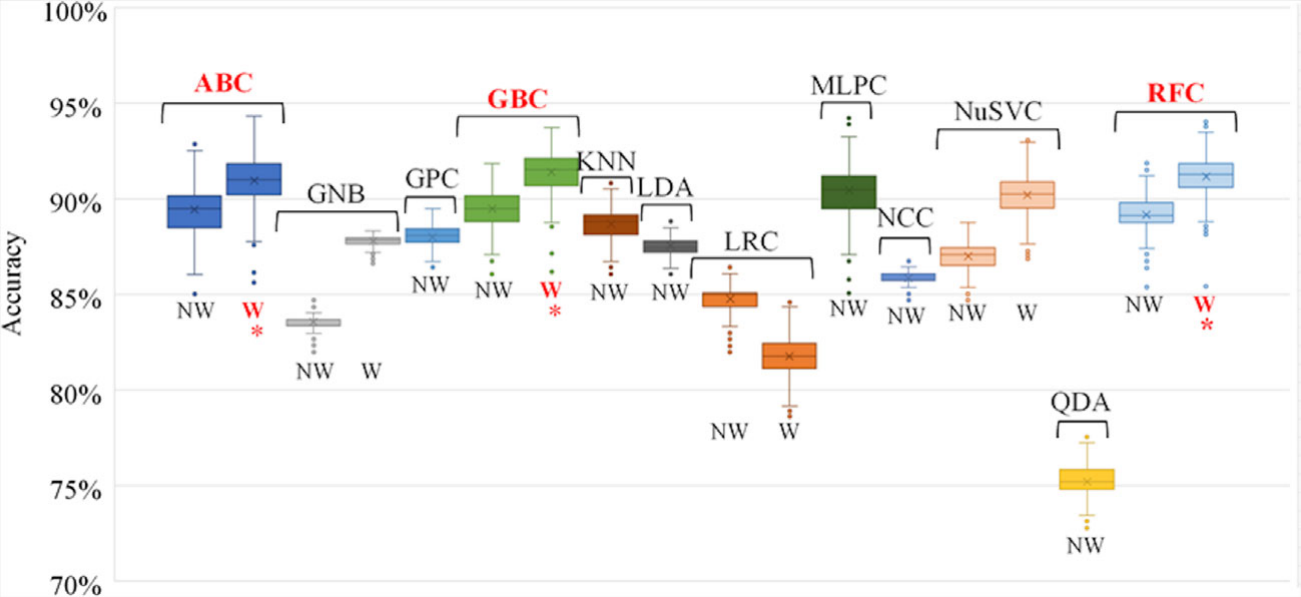
Individual model accuracy over 100 random training/testing iterations. For classification algorithms compatible with using weighted samples, both weighted and unweighted tests were performed. Final selected individual models are indicated with an *. NW, No Weight, W, Weighted, ABC, AdaBoost Classifier, GNB, Gaussian Naïve Bayes Classifier, GPC, Gaussian Process Classifier, GBC, Gradient Boosting Classifier, KNN, K-Nearest Neighbours Classifier, LDA, Linear Discriminant Analysis, LRC, Logistic Regression Classifier, MLPC, Multi-layer Perceptron Classifier, NCC, Nearest Centroid Classifier, NuSVC, Nu-Support Vector Classifier, QDA, Quadratic Discriminant Analysis, RFC, Random Forest Classifier.

The accuracy of each trained individual model is shown in **Figure 4**. In general, individual models that utilized weighted sample points to account for dosimetric plan quality (**Table 1**) were more accurate than those that did not. The highest performing classification algorithms were tree-based ensemble methods – AdaBoost (ABC), Gradient Boosting (GBC), and Random Forest (RFC) classifiers. These classification algorithms were used to train the individual models used in the subsequent voting model evaluation.

### Voting Model

A comparison between the performance of the individual models and the compiled voting model was performed to ensure that there was an improvement in performance when using the voting model. The top three weighted individual models, in terms of mean classification accuracy over the 100 iterations, were the AdaBoost Classifier (ABC), Gradient Boosting Classifier (GBC), and Random Forest Classifier (RFC). The results of the comparison are shown in **Table 4**. For all performance metrics there was an improved standard deviation for the voting model, illustrating more consistent model performance when using voting as opposed to the individual models. Additionally, for all metrics the voting model was as good or better than the results achieved when using only the individual model. This supports the use of the voting model over the individual models.

**Table 4:** Performance metrics for the top three weighted individual models (1^st^ = Gradient Boosting Classifier [GBC], 2^nd^ = Random Forest Classifier [RFC], 3^rd^ = AdaBoost Classifier [ABC]) and the voting model.

Performance Metric	GBC	RFC	ABC	Voting
Accuracy	91.6 ± 3.1%	90.4 ± 4.1%	89.5 ± 4.0%	92.2 ± 1.8%
Precision	88.0 ± 2.9%	84.8 ± 5.5%	86.6 ± 5.3%	87.8 ± 2.9%
Recall	93.4 ± 6.2%	95.5 ± 4.6%	89.9 ± 9.1%	95.5 ± 3.7%
F1 Score	90.6 ± 3.8%	89.7 ± 4.2%	88.0 ± 5.3%	91.4 ± 2.4%

Soft voting that predicts the class label based on the sums of the predicted probabilities was utilized.

### Feature Importance for Individual Models in Voting Model

**Figure 5:**
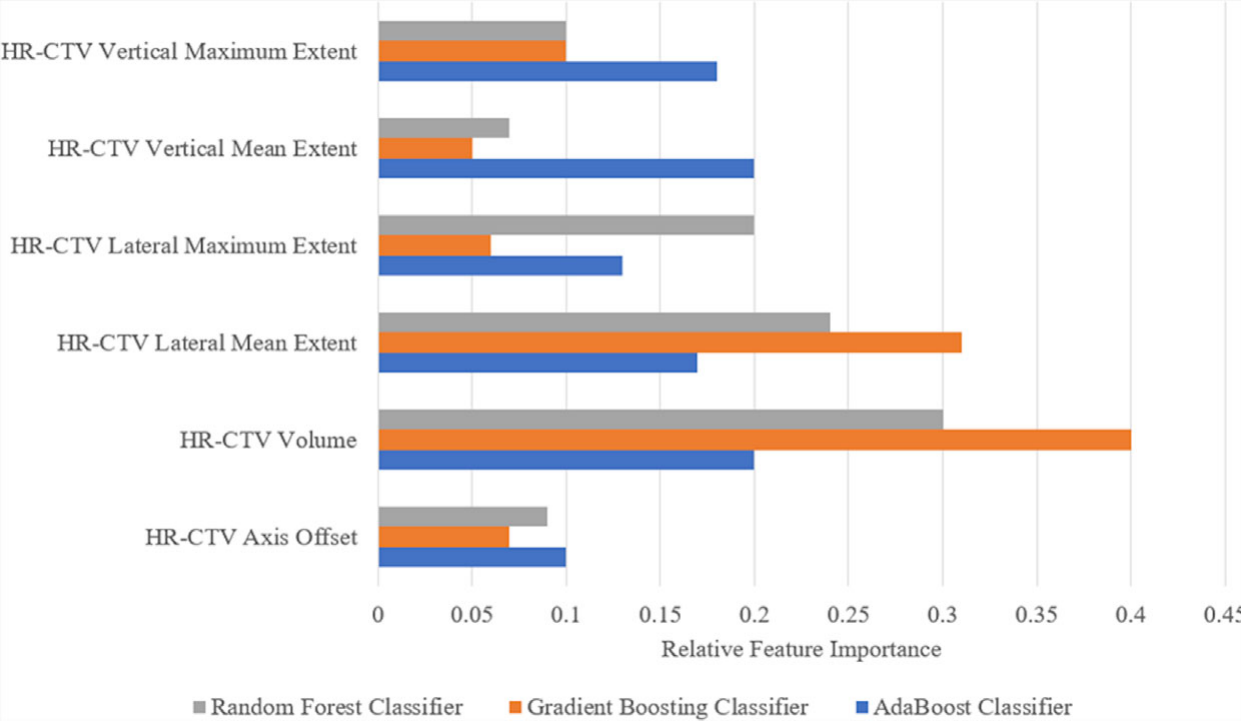
Feature importance for the six geometric features used in the three individual models selected for the final voting model. The more an attribute is used to make key decisions, the higher its relative importance. Importance is calculated by the amount that each attribute split point improves the performance measure (accuracy) of the algorithm.

**Figure 5** shows the relative feature importance of the subset of six HR-CTV features selected. For all individual models, the HR-CTV volume is the most important feature for the applicator selection process. For two of the three individual models the lateral mean extent of the HR-CTV the second most important feature.

### Voting Model Performance

Over the 1,000 test iterations, the voting model resulted in high scores for all metrics, which are reported in **Table 5**. Additionally, the LOOCV test resulted in an accuracy of 89.80% which is more reflective of true model performance when applied to a new sample.

**Table 5:** Performance metrics for voting model, calculated over 1,000 test iterations.

Performance Metric	
Accuracy	91.5 ± 0.9%
Precision	87.3 ± 1.1%
Recall	94.5 ± 1.7%
F1 Score	90.6 ± 1.1%
ROC-AUC	91.8 ± 1.0%
Leave-One-Out Accuracy (LOOCV)	89.80%

For each iteration, the KFold random state was set to the iteration number. The LOOCV accuracy is not calculated over these 1,000 test iterations as every data point acts as the test set once, meaning there are no random seeds that can be implemented.

## Discussion

In this work, we validated a voting ML model based on three classification algorithms with high predictive accuracy to be used as a preliminary decision-support tool validating applicator selection post-insertion in LACC brachytherapy treatments. An ML-based decision-support tool can provide planning assistance towards improving applicator selection quality and uniformity. The GEC-ESTRO group has identified the need for appropriate applicator and needle selection to deliver optimal dose distributions to the HR-CTV and normal tissues, which has been correlated with local recurrence and toxicity, respectively (35, 36). This work is the foundation of a prospective study that will evaluate the improvement in delivered dosimetry when using the software tool to support applicator selection

Feature selection methods were employed to identify the specific clinical features that contributed the most to the applicator selection process (**Table 3**). The identification of the HR-CTV lateral extent and volume as informative features supports the current applicator selection recommendations from the GEC-ESTRO group and the ABS, which confirm that HR-CTV geometry characteristics are a primary driver in needing hybrid interstitial needles (13, 14). During the feature importance investigation, it was also found that the HR-CTV lateral extent and volume held high feature importance for each of the individual models, further supporting the importance of these metrics in the applicator selection process.

The use of accuracy as a metric to compare models resulted in creating a voting model with high predictive capabilities. Using accuracy also provided the benefit of having an easily interpretable metric to express model performance. The use of a voting model was shown to increase model stability and provided a slight increase in performance above using the best performing individual model (**Table 4**). Additionally, the voting model displays a high recall (i.e., true positive rate) of 94.5%, indicating the model’s ability to identify the use of IS needles correctly. This shows that the model is less likely to incorrectly label a case where hybrid interstitial needles were required, minimizing the likelihood of the model suggesting an applicator that may lead to an underdosing of the target volume. Additionally, once trained the ML model is capable of predicting an applicator for an unseen patient in fewer than 10 s, making it a time-efficient process.

ML model performance can be limited by both poor-quality data as well as limited size of dataset, which will be a persistent issue in brachytherapy utilization. However, this was mitigated in this study by incorporating the dosimetric-based weighting factor to ensure the model was preferentially learning from the highest quality plans (**Table 1**). The performance metrics of the voting model demonstrate relative robustness and as a support tool, may be used in combination with clinical judgement to provide certainty when determining the need for interstitial needles. In the future, incorporating data from other cancer centres with large cervical HDR case volumes could increase the scope of the dataset, improving the quality of the decision-support tool by expanding the level of included expertise.

In its current form, the ML model utilizes post-insertion contours to extract the geometric features used by the model. This allows the model to act as a quality assurance (QA) tool, validating or contradicting the selection of applicator that was used clinically. For true prospective applicator prediction, an ethics-approved study will be conducted to apply this ML model to a dataset of contours extracted from pre-insertion diagnostic MR images. In this prospective study, the ML model will predict the use of an IC or IS applicator for a new patient based on the pre-insertion geometry metrics. The model prediction will then be compared to an expert’s prediction to test agreement and a consensus decision on which applicator to use for treatment will be made. This study will use simulated treatment plans to evaluate differences in dosimetric plan quality between the two applicators to assess the quality of decisions made by experts and the ML model. To effectively switch from post-insertion to pre-insertion images, the impact of the brachytherapy applicator on the deformation and displacement of the high-risk clinical target volume (HR-CTV) and other soft tissues must be evaluated. Previous research has investigated the impact of the brachytherapy applicator on soft tissue deformation, investigating both the volumetric variation and centroid displacement between pre-insertion and post-insertion brachytherapy CT images. Results indicated that although volumetric differences of the cervix-uterus were minor, displacements and deformations were observed (37).

## Conclusion

Using the open source Python ML package Scikit-Learn, this work establishes a framework for designing, training, and validating a preliminary ML-based tool to provide insight into the applicator selection process based on clinically relevant geometric features. Our results provide evidence that easily interpretable tree-based classification methods yield high discriminative performance. This work presents the first step in developing a comprehensive applicator selection tool and provides preliminary validation for the use of machine learning in the applicator selection process. Currently, the model can be used to validate a physician’s applicator choice post-insertion as a QA tool. Future work will extend this ML model for use in prospectively selecting brachytherapy applicators for the treatment of cervical cancer as well as optimal needle arrangements for treatment volumes that require interstitial needles. This prospective study has received ethics approval and utilizes the presented ML model trained on a dataset of pre-insertion geometry metrics. Improvement in the applicator selection process is expected to result in improved dose distributions that may yield improved treatment outcomes.

## Data Availability Statement

The raw data supporting the conclusions of this article will be made available by the authors, without undue reservation.

## Ethics Statement

The studies involving human participants were reviewed and approved by University of Calgary Health Research Ethics Board of Alberta Cancer Committee (HREBA-CC). Written informed consent for participation was not required for this study in accordance with the national legislation and the institutional requirements.

## Author Contributions

KS and PC collected data, extracted features, deployed and evaluated machine learning algorithms, interpreted and visualized results, and drafted the manuscript. MR and PM conceptualized the project and participated in the study design, analysis and interpretation of results, and drafting of the manuscript. RB and SY provided expert guidance throughout the study and interpretation of results. All authors contributed to the article and approved the submitted version.

## Conflict of Interest

Co-author MR has ownership interest in Okolo Health, a brachytherapy company.

The remaining authors declare that the research was conducted in the absence of any commercial or financial relationships that could be construed as a potential conflict of interest.
