# Corrigendum: Development of a Machine Learning Model for Optimal Applicator Selection in High-Dose-Rate Cervical Brachytherapy

**DOI:** 10.3389/fonc.2021.730375

**Published:** 2021-07-06

**Authors:** Kailyn Stenhouse, Michael Roumeliotis, Philip Ciunkiewicz, Robyn Banerjee, Svetlana Yanushkevich, Philip McGeachy

**Affiliations:** ^1^ Department of Physics and Astronomy, University of Calgary, Calgary, AB, Canada; ^2^ Department of Medical Physics, Tom Baker Cancer Centre, Calgary, AB, Canada; ^3^ Department of Oncology, University of Calgary, Calgary, AB, Canada; ^4^ Department of Biomedical Engineering, University of Calgary, Calgary, AB, Canada; ^5^ Department of Radiation Oncology, Tom Baker Cancer Centre, Calgary, AB, Canada; ^6^ Department of Electrical and Computer Engineering, University of Calgary, Calgary, AB, Canada

**Keywords:** gynecologic brachytherapy, intracavitary brachytherapy, high-dose-rate brachytherapy, radiation oncology, machine learning, decision-support tools

Philip Ciunkiewicz was not included as an author in the published article. The corrected Author Contributions Statement appears below.

KS and PC collected data, extracted features, deployed and evaluated machine learning algorithms, interpreted and visualized results, and drafted the manuscript. MR and PM conceptualized the project and participated in the study design, analysis and interpretation of results, and drafting of the manuscript. RB and SY provided expert guidance throughout the study and interpretation of results. All authors contributed to the article and approved the submitted version

In the published article, there was an error regarding the affiliation for Philip Ciunkiewicz. They should have ^4^Department of Biomedical Engineering, University of Calgary, Calgary, AB, Canada.

The authors apologize for these errors and state that this does not change the scientific conclusions of the article in any way. The original article has been updated

