# Supplementary material for: Development of a Machine Learning Model for Optimal Applicator Selection in High-Dose-Rate Cervical Brachytherapy
Source: Front Oncol. 2021 Mar 5;11:611437. doi: 10.3389/fonc.2021.611437 (PMC7973285; doi:10.3389/fonc.2021.611437)
Supplement: Supplementary file 1 [file Table_1.pdf]

TABLE S1. List of compared classifiers, their optimized hyperparameters, final optimized hyperparameter values, and compatability with using weighted samples when training the classifier.

Classifier	Abbreviation	Tuned Hyperparameters	Weighted Samples?
AdaBoost Classifier	ABC	*learning_rate = 1.4*<br>*n_estimators = 50*	Yes
Gaussian Naïve Bayes Classifier	GNB	*var_smoothing = 0.00000000001*	Yes
Gaussian Process Classifier	GPC	*max_iter_predict = 50*<br>*n_restarts_optimizer = 0*	No
Gradient Boosting Classifier	GBC	*learning_rate = 0.09*<br>*max_depth = 5*<br>*max_features = 3*<br>*min_samples_leaf = 15,*<br>*min_samples_split = 2*<br>*n_estimators = 55*<br>*subsample = 0.8*	Yes
K-Nearest Neighbours Classifier	KNN	*algorithm = kd_tree*<br>*leaf_size = 3*<br>*n_neighbours = 5*<br>*p = 1*	No
Linear Discriminant Analysis	LDA	*solver = svd*<br>*store_covariance = True*<br>*tol = 0.0000001*	No
Logistic Regression Classifier	LRC	*C = 0.01*<br>*max_iter = 90*<br>*penalty = l2*<br>*solver = newton-cg*<br>*tol = 0.00001*	Yes
Multi-layer Perceptron Classifier	MLPC	*activation = relu*<br>*alpha = 0.001*<br>*max_iter = 200*<br>*solver = lbfgs*<br>*tol = 0.00001*	No
Nearest Centroid Classifier	NCC	*metric = cosine*<br>*shrink_threshold = 0.00001*	No
Nu-Support Vector Classifier	NuSVC	*gamma = 1*<br>*kernel = rbf*<br>*nu = 0.45*<br>*shrinking = False*<br>*tol = 0.00001*	Yes
Quadratic Discriminant Analysis	QDA	*reg_param = 0.0000001*<br>*tol = 0.0000001*	No
Random Forest Classifier	RFC	*max_depth = 7*<br>*max_features = 2*<br>*min_samples_leaf = 2*<br>*min_samples_split = 2*<br>*n_estimators = 75*	Yes
